# Advanced Practice Nursing: “Training” Pillar in Supporting the Proposal in Brazil

**DOI:** 10.1590/0034-7167-2023-0118

**Published:** 2023-10-06

**Authors:** Cristina Maria Garcia de Lima Parada, Elisabete Pimenta Araujo Paz, Lucia Yasuko Izumi Nichiata, Dulce Aparecida Barbosa, Luciane Kantorski

**Affiliations:** I Universidade Paulista Júlio de Mesquita. Botucatu, São Paulo, Brazil; II Universidade Federal do Rio de Janeiro. Rio de Janeiro, Rio de Janeiro, Brazil; III Universidade de São Paulo. São Paulo, São Paulo, Brazil; IV Universidade Federal de São Paulo. São Paulo, São Paulo, Brazil; V Universidade Federal de Pelotas. Pelotas, Rio Grande do Sul, Brazil

**Keywords:** Advanced Practice Nursing, Public Policy, Health Postgraduate Programs, Nursing Education, Education, Nursing, Graduate, Enfermería de Práctica Avanzada, Políticas Públicas, Programas de Posgrado en Salud, Educación en Enfermería, Educación de Postgrado en Enfermería, Prática Avançada de Enfermagem, Políticas Públicas, Programas de Pós-Graduação em Saúde, Educação em Enfermagem, Educação de Pós-Graduação em Enfermagem.

## Abstract

**Objectives:**

to present the pillars that support what has been called Advanced Practice Nursing and discuss the necessary training for its implementation.

**Methods:**

elements contained in assessment documents for graduate programs proposals, reports of presentations by international professors in countries and selected scientific publications were gathered to compose the argument.

**Results:**

practice/competency (adds broad and in-depth knowledge about health processes and scientific evidence, clinical reasoning and clinical skills for therapeutic indications); 3) professional regulation (corresponding legislation and monitoring); and 4) funding (broad training and professional practice policy).

**Final Considerations:**

the agenda for implementing Advanced Practice Nursing in Brazil involves joining efforts to identify stakeholders for a work to legitimize their importance in the country’s health and education overview.

## RESUMO

**Objetivos:**

apresentar os pilares que sustentam o que vem sendo nominado como Enfermagem de Prática Avançada e problematizar sobre a formação necessária para sua implementação.

**Métodos:**

reuniram-se elementos constantes em documentos de avaliação de propostas dos programas de pós-graduação, relatórios de apresentações de docentes internacionais em países e publicações científicas selecionadas para compor a argumentação.

**Resultados:**

prática/competência (agrega conhecimento amplo e aprofundado sobre os processos de saúde e evidências científicas, raciocínio clínico e capacidades clínicas para indicações terapêuticas); 3) regulação profissional (legislação correspondente e acompanhamento); e 4) financiamento (política ampla de formação e de prática profissional).

**Considerações Finais:**

a agenda para a implementação da Enfermagem de Prática Avançada no Brasil passa por conjugar esforços na identificação de *stakeholders* para um trabalho de legitimação sobre sua importância no panorama da saúde e educação do país.

## RESUMEN

**Objetivos:**

presentar los pilares que sustentan lo que se ha denominado Enfermería de Práctica Avanzada y discutir la formación necesaria para su implementación.

**Métodos:**

se reunieron elementos contenidos en documentos de evaluación de propuestas de programas de posgrado, informes de presentaciones de profesores internacionales en países y publicaciones científicas seleccionadas para componer el argumento.

**Resultados:**

práctica/competencia (agrega conocimiento amplio y profundo sobre procesos de salud y evidencia científica, razonamiento clínico y habilidades clínicas para indicaciones terapéuticas); 3) regulación profesional (legislación correspondiente y seguimiento); y 4) financiación (política amplia de formación y ejercicio profesional).

**Consideraciones Finales:**

la agenda para la implementación de la Enfermería de Práctica Avanzada en Brasil implica aunar esfuerzos para identificar actores para un trabajo de legitimación de su importancia en el panorama de la salud y la educación del país.

## INTRODUCTION

The theme Advanced Practice Nursing (APN) is on the agenda in the nursing area in Brazil. Especially from 2015 onwards, articles published in national journals^(1-5)^ on the subject and the holding of numerous scientific events with APN as a central theme are identified.

Class and scientific entities have made efforts to discuss APN, defining the concept and scope, with emphasis in the Americas on the Pan American Health Organization (PAHO) and the American Association of Nurse Practitioners (AANP). In Brazil, the Federal Council of Nursing (COFEN), the PAHO Collaborating Center for human resources, located at the *Escola de Enfermagem de Ribeirão Preto, Universidade de São Paulo* (EERP-USP), and the Brazilian Association of Nursing, which constituted spaces of debate and working groups to enable such a discussion, stand out.

Despite the efforts undertaken, there are different understandings and operationalization of APN^(6-9)^. In the present text, the International Council of Nurses’ (ICN) understanding is adopted as a presupposition, in defending that, for professionals to be, in fact, recognized as developing APN, they need to acquire a knowledge base through specific training at the *stricto sensu* graduate level, at least a master’s degree. Nurses with this training have decision-making skills and clinical skills for expanded practice, whose characteristics are determined by the context and/or the country in which they are accredited^(10)^. By taking over this position, it is not disregarded that nurses at different points in the care network, in different settings and spaces, are producers of relevant and innovative practices through the activities they carry out, based on the health system advancement in the country.

The considerations presented here are the result of the work group’s (WG) discussion constituted by the Nursing Assessment Area Coordination (2018 to 2022 management) of the Coordination for the Improvement of Higher Education Personnel (CAPES - *Coordenação de Aperfeiçoamento de Pessoal de Nível Superior*), represented by PhD: Cristina Maria Garcia de Lima Parada; by the academic coordination assistants: Luciane Kantorski; professional Lucia Y. Izumi Nichiata; in addition to PhD as Dulce Aparecida Barbosa (*Universidade Federal de São Paulo* - UNIFESP/Brazilian Nursing Association), Elisabete Pimenta Araújo Paz (*Universidade Federal do Rio de Janeiro* - UFRJ/Federal Nursing Council) and Beatriz Toso (*Universidade Estadual do Oeste do Paraná* - UNIOESTE/APN Latin Network), and the last professor stayed in the group until the end of 2021. Considering WG’s discussions held and understanding on the subject, this article aims to present the pillars that should support APN, highlighting the necessary training and the challenges to its implementation.

### Founding Pillars of Advanced Practice Nursing

It is possible to identify the beginning of what has been delineated as APN, from the post-World War II period, especially in the United States of America (USA) and Canada. In the first case, with the expansion of North American nurses’ autonomy, especially in the area of public health, including because there is little medical participation in this field, the expansion of the scope of their practices has grown and developed. One of the first graduate courses in the USA for nurses, from the perspective of APN, took place in 1967, in the area of child care in the community. The care produced by nurses with this training proved to be very relevant, even demonstrated in a large study that recommended APN to the entire population^(11)^. Favorably at the time, for recognition of APN, the US Department of Health, Education and Social Welfare committee’s conclusions pointed to clinical practice as the main objective of APN’s function, involving direct and indirect care for patients and their families, groups, communities or populations. Based on this social recognition, the necessary training and specific registration for nurses who perform extended practice with formal training were defined^(11)^.

Thus, during the 1970s, the term “APN” began to be used in the USA, delimiting four fields or domains of practice: anesthetist nurse, nurse-midwife, clinical nurse specialist and nurse practitioner. Courses proliferated, resulting in the recognition of a new nursing model, culminating in the 1990s with profiles of nurses who, having completed master’s or doctoral studies, developed their scientific and professional functions within APN^(11)^.

**Figure 1:**
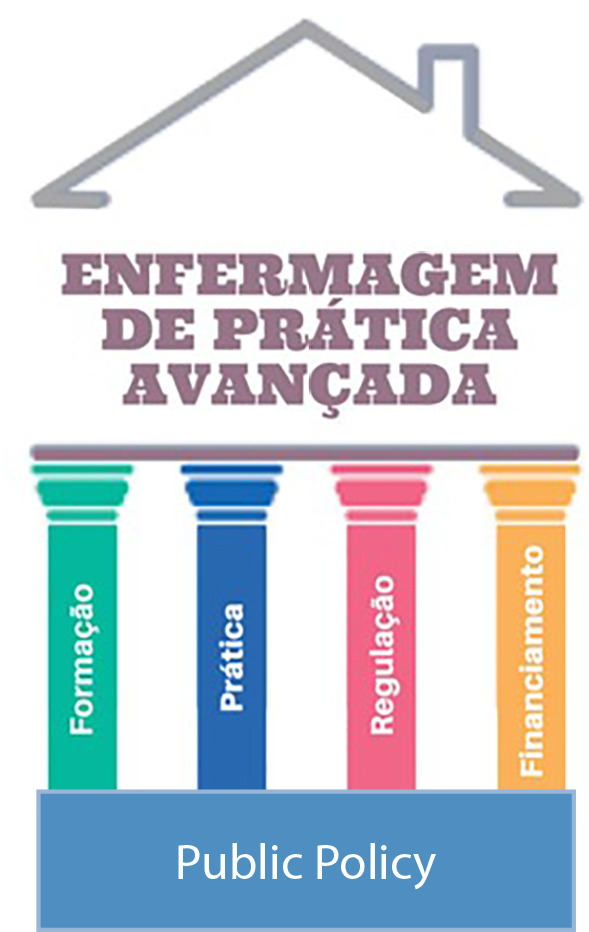
Graphical representation of the basis and supporting pillars of Advanced Practice Nursing

*Source: based on the International Council of Nurses^(8)^.*

In Canada, in the 1960s, APN emerged as a social response to the population’s needs and with a strong educational nature, preparing two types of professionals for broader work. First, Nurse Practitioners (NP) were formed in response to the shortage of primary care physicians in rural and remote areas, and currently carry out specific actions to complex patient care needs and develop quality assurance and leadership programs and activities, with initiatives to promote practices based on scientific data, noting significant numerical growth only at the beginning of this century^(12)^.

The second group of professionals, Clinical Nurse Specialists (CNS), started working in hospital environments at the end of the 1960s, with the aim of improving the quality of nursing services, in response to the increase in specialization and technology^(13)^. While CNS have expertise in a clinical nursing specialty and their role includes practice, consultation, education, research and leadership, NP have expanded their clinical functions and have legal authority to carry out additional activities, usually carried out by physicians, i.e., diagnosing, ordering tests and prescribing medications, working in the community in settings such as community health centers, family physicians’ offices, primary care networks and long-term care^(14)^.

In recent decades, APN began to expand globally so that in 2016 its presence was identified in 19 countries, however using different denominations, such as Advanced Nurse Practitioner, Advanced Practice Nurse and Specialist Nurse, among others, in addition to different domains of practice (NP and CNS) and performance scenarios (Primary Health Care, hospitals and medical)^(6)^. A growing trend was identified in the last decade of scientific publications on APN; however, analyzing the studies, it was found that they do not clearly and detail about regulation of work, professional training and impacts of these practices in the field of nursing itself^(15)^.

The understanding of APN is that it is not a generalist practice, but an advanced one, performed autonomously, in the sense of having decision-making skills and clinical competences. As a professional profile, it is expected to have abstract critical reasoning, which includes initial assessment of users, families and communities under their care about the health problems that affect them, the effective indication of therapeutic alternatives and follow-up through monitoring and back-assessment. It can be added that this practice transcends following nursing protocols, elaborated within the scope of national or local health policies^(16)^.

Taking the ICN recommendations, APN must integrate research, education, care practice and management, and its actions must be based on scientific evidence. Moreover, for its development, it is necessary to consider training, technical-scientific skills and professional regulation^(8)^. Thus, its implementation has occurred in different countries through specific public policies and changes in legislation and professional regulation, transforming practice scenarios^(17)^.

PAHO recognizes APN as an important strategy for expanding access to health in Latin American and Caribbean countries, having approved, in September 2013, Resolution CD52.R13, which guides countries to prioritize public policies in Primary Health Care (PHC) to qualify their workforce and increase the number of advanced practice nurses, to support health systems organized in PHC^(18-19)^.

The WG understands that APN implementation is primarily based on the country’s public health and education policy. Necessarily, the group considers that the proposal’s support pillars are: 1 - graduate training, with strong content that actually responds to the expected professional profile; 2 - practice/competency, which adds broad and in-depth knowledge about health processes and scientific evidence, clinical reasoning for decision-making and clinical skills for therapeutic indications; 3 - professional regulation, with corresponding legislation and follow-up; and 4 - funding, which covers a broad policy for training and professional practice.

The four pillars are briefly presented, however this article reflects in more depth on the first pillar, training, having as a reference the *stricto sensu* graduate degree in nursing, as proposed by the ICN^(8)^.

### “Training” Pillar

The combination of graduate and clinical experience allows nurses to develop the necessary skills in APN^(13)^. Certainly, a nurse with a master’s degree qualification for advanced practice could operate in education, leadership, management, research and clinical practice. In the Cycle of International Lectures on Advanced Nursing Practices, promoted by COFEN, PAHO Brazil and the Collaborating Center for Research in Nursing at EERP-USP in 2021, PhD Denise Bryant-Lukosius spoke about training experiences in Canada, where most programs require at least two years of experience in nursing practice before starting the master’s program in APN and where, on average, about 750 clinical hours are incorporated into training. The researcher pointed out that, in Ontario, the professional master’s program of six academic institutions, including McMaster University, developed integrated programming of essential subjects, combining online and face-to-face activities with an emphasis on increasing levels of clinical and diagnostic assessment, and developing leadership skills and other content or domains of advanced practice, such as research, evidence-based practice, quality improvement projects and clinical evidence, among other topics^(20)^. This partnership has been important in enabling the training of nurses in the context of APN.

In Brazil, the *stricto sensu* graduate course in nursing, a professional modality, began in 2002, and the professional doctoral degree in the country dates from 2019. However, this modality was already foreseen in the well-known Opinion *Sucupira*, from 1965, since this document, which guided the development of Brazilian graduate programs, already indicated the distinction between academic and professional programs^(21)^.

Professional education is defined as that which involves training for transforming professional practice, through the incorporation of the scientific method. It is aimed at the public, preferably from outside academia, and is intended for the management, production and application of knowledge oriented towards research applied to problem solving, proposing new technologies and technological improvement^(22-24)^. In nursing, it is considered that it is focused on training professionals, whose training takes place through the production and consumption of the best evidence for carrying out practices in the study of techniques, processes or themes that meet some social demand^(25)^.

In December 2022, the nursing area had 24 graduate programs, of which 21 had master’s courses and three had master’s and doctoral courses, two of which started in 2019 and one still to be implemented^(26)^. These programs, approved or in operation, did not include APN proposals, as understood by this group.

Proposals for new courses in the professional master’s degree modality must present a curricular structure that emphasizes the articulation between up-to-date knowledge, mastery of relevant methodology and application oriented towards specific professional field of activity. For this, a portion of the staff must be made up of professionals recognized in their areas of knowledge for their qualification and outstanding performance in the field relevant to the course proposal. The course’s final work must always be linked to real problems in professional-student’s area of activity and according to the nature of the area and the purpose of the course, and can be presented in different formats^(27)^.

As a result of the WG that discussed APN, the considerations on what should be considered in Presentation of Proposals for New Courses (APCN) of a professional master’s degree in nursing in Brazil with a focus on APN follow, emphasizing that, as it is a first experience, it is not considered pertinent to present proposals of doctoral course. In elaboration, emphasis is placed on what the area considered in a publication on APCN^(25)^ and studies that support the understanding of APN. Thus, it is indicated that it be considered in the proposal preparation:

- Explanation of teaching and research infrastructure focused on APN, with emphasis on health services/institutions where the hours of clinical practice will be carried out;
- Course proposal presentation addressing: indication as to the history and context of the proposal; adaptation to the development plan of the proposing Higher Education Institution and self-assessment policy; clear objectives regarding APN competencies; coherence between area of concentration (AC), lines of research or action (LR/A), research projects (RP) and/or technological development and innovation projects (TDIP) and curriculum structure, including specific subjects and bibliographic references in APN.

It should be noted that the APN proposal is not limited to the offer of isolated subjects and without coherence with the whole, and that it should be explicit how the 500-600 hours of clinical practice will be developed and how the multidisciplinary experience will be incorporated, considering the average training time of 24 months for the master’s degree;

- Need for clarity regarding the statement of student selection criteria - nurses with at least three years of experience in health services;
- Indication as to the number of vacancies and ratio of advisees per advisor, with the warning that in the first year a maximum of two students should be provided per advisor in the course;
- Explanation regarding the intended training and profile of the graduate, considering that it is a professional course. This aspect should be crucial in the proposal, and the list of expected competencies aimed at clinical autonomy and expansion of practices should be presented;
- Presentation of the course statute and the implementation plan of the program’s self-assessment policy;
- General characterization of the teaching staff with compliance with the APN proposal. This is another crucial aspect in offering a master’s course in APN in order to have this specialty in the country, considering that both professors and field preceptors must have a broad command of clinical practice, with proven experience in the care area^(26-27)^;
- The minimum number of permanent professors (PP) for the proposed new course is 12. At least 60% of them must have undergraduate and/or graduate training in nursing and at least 70% must have a full-time relationship with the proposing institution (40 hours). In addition to the contract workload at the proposing educational institution, the proposed program’s dedication must be made explicit;
- In PP description, the diversification in the origin of training, performance in nursing or related areas, professional experience compatible and adequate to the course proposal and national and international projection must be made explicit;
- Up to 20% of the PP staff can be made up of professionals with recognized experience in the proposed field, even if they do not have a doctor’s degree. These professionals, however, must have the same level of integration and academic production as PhD PP;
- PP that make up the proposal may be linked as a PP in up to three programs, provided that participation in three programs does not exceed 30% of professors in the proposal. When a professor from outside the proposing institution participates in the PP framework, approval from the institution of origin must be presented, whenever the professor is hired on an exclusive dedication basis (40 hours);
- PP should be responsible for most guidance, teaching and research activities. Collaborating’s and visiting professors’ participation should not characterize dependency, nor be used to meet the minimum requirements of scientific production. The number of collaborating professors must be limited to a maximum of 20% of PP. The intellectual production of visiting professors and collaborators will not be considered for assessing compliance with the minimum requirements for production and scientific maturity;
- PP must demonstrate experience in adequate guidance in quantity, quality and regularity, meeting the requirements of the area for the teaching profile;
- All PP must present, at least, completed orientation of undergraduate final theses or scientific (SI) and/or technological initiation. At least 80% of PP must present completed master’s orientation;
- At least 80% of PP linked to the proposal must demonstrate joint work for at least one year at the proposing educational institution and present intellectual production relevant to LR/A AC, demonstrated by their scientific, bibliographical and/or technical production, which must be equally distributed among the PP. Likewise, professors’ experience and compliance to teach the subjects that support the proposal will be analyzed. All PP must be linked to at least one technical and technological production.
- It is necessary to explain the follow-up policy for course professors (accreditation, re-accreditation and de-accreditation) and students;
- PP’s qualified intellectual production and the proposed course must be presented, considering the position of the journals in the Web of Science, Scopus or H5-Google databases and auditable technical and technological production, according to the document “*Considerações sobre Classificação de Produção Técnica e Tecnológica* (PTT)”^(28)^.

### “Practice/Competency” Pillar

In 2014, the PAHO Executive Committee proposed Universal Health Coverage (UHC), or Strategic Plan for Universal Health Coverage. To achieve UHC, the importance of developing high quality PHC services and the need to invest in human resources, especially in the training of nurses so that they can carry out these services. This strategic plan was drawn up based on a 2013 PAHO resolution, and is focused on strengthening the PHC workforce through expanding the introduction and numbers of APN^(29)^.

### “Regulation” Pillar

There is still no APN regulation in Brazil, a condition that depends on important political articulation with the Ministry of Health (MoH) and Ministry of Education. There were initiatives with the MoH and the Department for Management of Work and Health Education (SGETS - *Secretaria de Gestão do Trabalho e da Educação na Saúde*) in this regard, but negotiations have stalled in recent years, paralyzing any initiatives to foster debate at the federal level. It is necessary to advance in this item, with a review of legal parameters and dissemination of advanced practice in the country with universities, professional associations, local governments, unifying concepts and professional skills for regulation, in order to overcome challenges and problems in identifying legal barriers that can significantly delay the advancement of this activity in the country. It is also important to verify whether the routines in the units can be revised so that it becomes necessary to expand the autonomy of professional actions, including investing in work to clarify colleagues about the differentiated role of APN.

In the United Kingdom, with several years of advancement in APN, at different levels of health care, it is no different. They identify challenges with APN regulation, which include definition and variations in scope of practice, organizational constraints, and lack of support for implementation. These challenges are exacerbated by a lack of clarity about precisely the role played, indicating the need to improve the regulation of this practice. UK nurses recognize that APN role lacks consistency in scope of practice, training and regulation. In experience, professional practice is regulated by one of three different bodies, nationally by central government or a professional body, or locally by employers. The Royal College of Nursing has responded to these challenges by introducing ‘accreditation’, a system of recording qualifications, skills and experience, but the adoption of this process remains to be assessed^(30)^.

In Brazil, political meetings are already on the agenda of educational programs linked to municipalities and professional master’s degrees, but unified action is still needed to insert APN in the national negotiation with policy makers, inserting it into the Brazilian National Policy on Human Resources in Health. The regulatory components are what will allow, from APN, nurses to be able to prescribe medication, refer patients to other services, request diagnostic tests that make them independent, these being clearly defined; therefore, qualified training with sustained clinical evidence must persist. Certification and periodic authorization from specific bodies or associations may be necessary, and legislation protects titles obtained in professional master’s degrees, as in other countries.

As there is no legislation/regulation in Brazil for this particular practice to be carried out by nurses, while its implementation in the country is being discussed, it is necessary to move towards establishing APN as a policy for the development of human resources for the Brazilian System of Health (SUS - *Sistema Único de Saúde*).

### “Funding” Pillar

This is an essential pillar and perhaps the greatest challenge for APN implementation in Brazil, since funding is not only related to training, but also to the other support pillars discussed here. Precisely, this is a pillar that has not been highlighted in discussions and publications on APN in Brazil. Without intending to go deeper, this text discusses some issues considered relevant.

DiCenso et al. (2010), analyzing the development of APN in Canada in the 40 years prior to the publication of the study, demonstrate the impact it had on improving health care quality in the country. They reaffirm the need for funding sustainability in the set of public policies, with the existing legal understanding that the State must be the provider as well as the importance of valuing workers’ remuneration for action at a national level^(31)^.

It must be remembered that, in Brazil, the SUS is historically and socially marked by structural financing problems^(32)^. In the country, investments in health are around 9% of the Gross Domestic Product (GPP), but of this amount, only 46% correspond to public spending^(33)^. The situation of underfunding is aggravated when considering fiscal and tax waivers^(34)^.

The effort to build a universal public system failed to increase the share of public spending, which is lower than private spending via health coverage plans and carried out directly by families. APN needs to be understood in this context, and the very uneven composition of the nursing team in the country must be considered: 77% are technicians and assistants, while 23% are nurses, gathered predominantly in the large urban centers of the capitals, with a concentration in the Southeast region and a shortage in the North and Northeast regions^(35)^. It is alerted that, both in the world and in Brazil, social rights, especially of workers, ultimately depend on structural changes^(36)^.

In implementing the APN, it is also necessary to problematize that *stricto sensu* graduate studies in the country have also been suffering an accelerated process of public defunding, with a significant reduction in the volume of resources destined for research and graduate scholarships. The privatization of this educational level is noticeable, with the diversification of institutions and courses over the years. The few financial resources invested in public education end up resulting in a low value for individuals of educational age, when comparisons are made between Brazil and several countries^(37)^.

It is also important to remember that, within the scope of graduate programs, in the professional modality, there is no funding for scientific-technological and innovation projects in the form of funding or scholarships from the main research agencies, such as the Brazilian Council for Scientific and Technological Development (CNPq - *Conselho Nacional de Desenvolvimento Científico e Tecnológico*) and state Research Support Foundations (FAP - *Fundações de Apoio à Pesquisa*). Despite these aspects, it is necessary to highlight the progress in the area of nursing, particularly in professional programs, noted from funding notices through a partnership agreement between CAPES and COFEN, since 2017. With the resources invested, it is possible to identify improvement in the training of masters and consequent qualification of practices resulting from research, innovation and technological development projects, with examples of relevant social impact^(38)^.

### Challenges to implementing Advanced Practice Nursing in Brazil

Based on the steps and strategies suggested for implementing APN in Latin America and the Caribbean^(39)^, barriers to be overcome in Brazil are identified, such as those discussed below:

1. Lack of clarity about the work performed by nurses in the particular scope of APN, including among professionals themselves, but also by universities and education, health and work management institutions. At this point, in order to overcome these barriers, a broad discussion is needed to advance the proposal, in the sense of even adopting it as an important starting point towards SUS improvement and incorporation of these professionals into the system. It is worth considering that the change in the alignment of an academic training of advanced practice nurses is centered on several issues, and, among one of the most important, is the preparation of these specialists at the master’s level, with an emphasis on subjects of a practical assistance nature, in which interprofessional training is relevant in preparing for the expansion of their professional skills, as shown by research with APN.
2. Weaknesses in the implementation of professional master’s programs, collaborative networks and research environments, technology and innovation in nursing and health, with the goal of safe and excellent care.
3. From the point of view of training, it is necessary to think about modifying professional graduate programs’ curricular structure, safeguarding the principles that structure the APN. This approach is important since graduation, as there is, in fact, APN implementation.
4. Medical professionals’ resistance to the proposal implementation, based on the Medical Act Law.

It is noteworthy that, in a recent experience in Chile, when outlining the nine steps and progress made in the country to implement the APN, based on the Participatory Evidence-based Patient-focused Process (PEPPA) approach, the importance of stakeholders in the perspective of existing power, whether visible or not, for increasing the possibility of successful APN implementation. They highlight the involvement of various actors, interested in the PHC theme, to discuss APN concept and role; the Chilean APN-PHC network’s work, led by the only coalition of the academic world of Chilean nursing and the Commission on Human Resources in Health of the Brazilian National Cancer Plan; in addition to the clear collaboration of the Ministry of Health, the Municipal Health Councils and the Brazilian National Association of Nurse Practitioners^(40)^.

Thus, the agenda for APN implementation in Brazil involves joining efforts in identifying stakeholders for a work of legitimizing its importance in the country’s health and education overview, not only crediting advanced practice nurses the task of correcting/solving access inequalities and inequities present in the SUS.

The effective APN implementation must be aligned with the 2014-2024 Brazilian National Education Plan^(41)^, the 2021-2030 Brazilian National Graduate Plan, which is under construction, and the Brazilian National Health Policy. The country has already taken the first steps towards the national discussion of advanced specialist nurses who need it; however, such an initial discussion needs to gain breadth among those who can give credibility and recognition to quality clinical training, strong scientific bases and clinical experiences that support the incorporation of new skills, notably in PHC.

It is also necessary to consider that forming a nursing specialty at the national level requires the availability of budgetary resources for investment in faculty and field preceptors with expertise and autonomy to support advanced clinical training, in addition to incorporation of research results for health teams. Costs and returns to the health system must constitute a policy aligned with the profile of professionals that the country needs. Overcoming regional differences regarding job supply in the country, autonomy to prescribe a set of medicines and treatments, incorporation of technologies, scientific leadership, interprofessional training are still challenges that need to be on the agenda of political negotiations of associations and councils professionals, MoH and Ministry of Economy.

These are some of the first challenges to be overcome for APN implementation. Let us continue on the path.
